# Aberrant intrinsic neural network strength in individuals with “smartphone addiction”: An MRI data fusion study

**DOI:** 10.1002/brb3.2739

**Published:** 2022-08-31

**Authors:** Mike M. Schmitgen, Nadine D. Wolf, Fabio Sambataro, Dusan Hirjak, Katharina M. Kubera, Julian Koenig, Robert Christian Wolf

**Affiliations:** ^1^ Department of General Psychiatry, Center for Psychosocial Medicine Heidelberg University Heidelberg Germany; ^2^ Department of Neuroscience, Padua Neuroscience Center University of Padova Padua Italy; ^3^ Department of Psychiatry and Psychotherapy, Central Institute of Mental Health, Medical Faculty Mannheim Heidelberg University Mannheim Germany; ^4^ Department of Child and Adolescent Psychiatry, Psychosomatics and Psychotherapy University of Cologne, Faculty of Medicine and University Hospital Cologne Cologne Germany

**Keywords:** addiction, brain activity, data fusion, gray matter volume, MRI, smartphone

## Abstract

**Background and objectives:**

Excessive smartphone use, also referred to as “smartphone addiction” (SPA), has increasingly attracted neuroscientific interest due to its similarities with other behavioral addictions, particularly internet gaming disorder. Little is known about the neural mechanisms underlying smartphone addiction. We explored interrelationships between brain structure and function to specify neurobiological correlates of SPA on a neural system level.

**Methods:**

Gray matter volume (GMV) and intrinsic neural activity (INA) were investigated in individuals with SPA (*n* = 20) and controls (*n* = 24), using multimodal magnetic resonance imaging and multivariate data fusion techniques, that is, parallel independent component analysis.

**Results:**

The joint analysis of both data modalities explored shared information between GMV and INA. In particular, two amplitudes of low frequency fluctuations‐based independent neural systems significantly differed between individuals with SPA and controls. A medial/dorsolateral prefrontal system exhibited lower functional network strength in individuals with SPA versus controls, whereas the opposite pattern was detected in a parietal cortical/cerebellar system. Neural network strength was significantly related to duration of smartphone use and sleep difficulties.

**Discussion and conclusions:**

We show modality‐specific associations of the brain's resting‐state activity with distinct and shared SPA symptom dimensions. In particular, the data suggest contributions of aberrant prefrontal and parietal neural network strength as a possible signature of deficient executive control in SPA.

**Scientific significance:**

This study suggests distinct neural mechanisms underlying specific biological and behavioral dimensions of excessive smartphone use.

## Graphical abstract

We explored interrelationships between brain structure and function to specify neurobiological correlates of "smartphone addiction" (SPA) on a neural system level. The data suggest contributions of aberrant prefrontal and parietal neural network strength as a possible signature of deficient executive control in SPA.

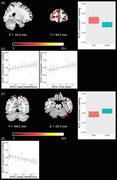

## 1 INTRODUCTION

In the past years, negative physical and psychosocial effects associated with excessive smartphone use have been emphasized by a growing number of studies (Demirci et al., 2015; Duke & Montag, 2017; Grant et al., 2019). Recent research highlighted behavioral similarities between excessive smartphone use and other addictive disorders, such as failure to resist use, withdrawal, continuation of use despite being aware of negative consequences, or deception of others regarding the amount of time spent on use (Lin et al., 2016). Moreover, excessive smartphone use has been repeatedly linked to impulsiveness and depression (Demirci et al., 2015; Grant et al., 2019). These behaviors also show close similarity to the criteria for internet gaming disorder (IGD) in the DSM‐5 (Petry et al., 2015). Consequently, criteria for “smartphone addiction” (SPA) were introduced (Lin et al., 2014) to define a condition characterized by excessive smartphone use and its problematic consequences for work‐related achievements and interpersonal relationships, as well as for physical and mental health (Demirci et al., 2015; Duke & Montag, 2017; Grant et al., 2019).

Although the term “smartphone addiction” has been anchored in several validated psychometric instruments, for example, the Smartphone Addiction Scale (SAS) (Kwon, Lee, et al., 2013) or the Smartphone Addiction Inventory (SPAI) (Lin et al., 2014), it has been criticized for conceptual and taxonomic reasons. In this regard, and because of possibly being a mobile branch of gaming disorder, alternative terms (e.g., “smartphone use disorder” or “excessive smartphone use”) were suggested (Montag et al., 2019), and it was argued that research should focus more on negative consequences of SPA instead of pursuing the question, if it should be considered as a behavioral addiction (Wacks & Weinstein, 2021). We acknowledge that the proposed alternative terms would be more appropriate, but we use the term SPA in accordance with the terminology of the psychometric instruments used in this study. Addictive use of specific internet applications has been recently addressed in the context of the Interaction of Person‐Affect‐Cognition‐Execution (I‐PACE) model (Brand et al., 2016), which combines psychological and neuroscientific theories of addictions. According to this model, addictive behavior emerges from the interplay of personal characteristics and moderating and mediating variables, such as brain volume or neural activity (Brand et al., 2016). For instance, in IGD, distinct brain areas have been shown to be involved, particularly anterior cingulate cortex (ACC), dorsolateral prefrontal cortex (DLPFC), and precuneus (Yao et al., 2017). Similarly, individuals with SPA may exhibit structural and functional changes at the neural structural and functional level (Horvath et al., 2020), such as reduced gray matter volume (GMV) or intrinsic neural activity (INA) in ACC (Horvath et al., 2020), altered functional connectivity at rest (Kim et al., 2016), and changes in neural activity in ACC and frontoparietal cortices during affective processing (Chun et al., 2017).

Of note, most research in technology‐associated behavioral addictions focused on either brain structure or function. To our knowledge, no study so far has provided combined information on both modalities in the same sample of affected individuals. While modality‐specific descriptive approaches are clearly useful, such attempts may disregard important sources of joint information between modalities. Such information is essential for directly detecting similarities among patterns of brain structure and function within and between specific populations. Data fusion approaches based on multivariate statistical techniques take advantage of a joint examination of various information sources of the same sample at the same time and are able to detect cross‐information of data, that is, co‐altered patterns of brain changes that may be partially missed in conventional separate analyses (Sui et al., 2012). Here, we used parallel independent component analysis (p‐ICA), a multivariate method to capture joint information from two data sources (Pearlson et al., 2015), to multimodally expand our previous descriptive findings of regionally abnormal GMV and INA in SPA (Horvath et al., 2020) by exploring joint information between these modalities, which cannot be fully detected by conventional mass‐univariate statistical approaches (Sui et al., 2012). To this end, p‐ICA was used to explore interrelationships between structural and functional networks, as well as relationships between network strength and psychometric scores indicating distinct behavioral dimensions of SPA. GMV and INA, in terms of the amplitude of low frequency fluctuations (ALFF), were entered the p‐ICA analysis. ALFF captures the relative magnitude of blood oxygen level dependent signal changes of intrinsic neural activity in distinct brain regions. This approach has been shown to be suitable to identify brain regions/networks with aberrant local functioning in patients with substance‐use disorders and IGD (Liu et al., 2018; Yuan et al., 2013).

We expected to find independent components showing differences between individuals with SPA and controls, that is individuals showing nonaddictive smartphone use, which depict hidden factors, which were not revealed by the former approach of separately analyzing GMV and ALFF data (Horvath et al., 2020). Furthermore, we explored associations between such hidden factors and distinct SPAI‐I dimensions, as suggested by a recent confirmatory factor analysis, that is, “time spent,” “compulsivity,” “daily life interference,” “craving,” and “sleep interference” (Pavia et al., 2016).

## 2 MATERIALS AND METHODS

### 2.1 Participants

In this secondary analysis, we used a subsample of participants referred to by Horvath et al. (2020) to take advantage of multivariate fusion techniques for multimodal data that allow the investigation of functional network strength. Individuals were recruited using flyers and posters distributed at Heidelberg University campus and city center and via ads on social media platforms undinger. A total of 132 persons expressed their interest in the study. After applying inclusion criteria (i.e., sufficient German language skills, right‐handedness, age 18–30 years, no general contraindications for magnetic resonance imaging [MRI] or self‐reported neurological or mental illness, no IGD, score on the short‐form Internet Gaming Disorder Scale [IGDS‐sf] <6), data from 44 participants (matched for age and gender) were used for final analyses. We defined two user groups based on the short version of the SAS (SAS‐SV), where cut‐off values of >31 for males and >33 for females were used to define a group of excessive smartphone users (SPA; *n* = 20, 14 females) (Kwon, Kim, et al., 2013). Participants below the cut‐off score were assigned to a group of controls (n‐SPA; *n* = 24, 17 females).

SAS‐SV and IGDS‐sf were used during a prescreening process to assign interested persons to one of the two defined groups (either SPA or n‐SPA; SAS‐SV) and to exclude persons showing IGD (IGDS‐sf) to prevent potentially biasing effects of IGD on the collected data. Before MRI, participants completed the SPAI, the Beck Depression Inventory (BDI)‐II (Beck et al., 1961), and the Barratt Impulsiveness Scale (BIS) version 11 (Patton et al., 1995). The SPAI is a comprehensive scale that measures a wide range of addictive behaviors related to smartphone use (Lin et al., 2014). Recently, a confirmatory factor analysis of the SPAI, SPAI‐I, suggested a five‐factor model of the SPAI, which showed better fit in a European population than the original SPAI (Pavia et al., 2016). For the purpose of this study, SPAI scores were recalculated according to the SPAI‐I. The following factors were considered: (1) time spent, consisting of four items capturing the difficulty of stopping and devoting more time and resources to use the smartphone. (2) Compulsivity, consisting of four items involving the degree of discomfort and emotional distress when being deprived of using the smartphone and not stopping use in spite of negative consequences. (3) Daily life interference, consisting of eight items describing the interference of smartphone use with other daily activities and interpersonal problems due to smartphone use. (4) Craving, consisting of five items capturing the degree of being unable to resist the urge to continue the behavior, and (5) sleep interference, including three items focusing on the relationship between smartphone use and sleep duration/disturbance (Pavia et al., 2016). For completeness, SPAI scores were also calculated following the four‐factor solution offered by Lin et al. (2014), that is, (1) compulsive behavior, (2) functional impairment, (3) withdrawal, and (4) tolerance (Lin et al., 2014). The BDI and BIS questionnaires were used to assess for depressive symptoms and impulsive personality traits, respectively, as depression and impulsiveness have been previously linked to excessive smartphone use and behavioral addictions (Demirci et al., 2015; Grant et al., 2019, 2010).

The study was approved by the Ethics Committee of the Medical Faculty at Heidelberg University and carried out in compliance with the Declaration of Helsinki. All participants gave written informed consent prior to inclusion in the study. All participants received monetary compensation (30€) for their participation.

### 2.2 MRI data acquisition

A 3‐T Magnetom TIM Trio MR Scanner (Siemens, Erlangen) equipped with a 32‐channel head coil was used to collect whole‐brain structural and functional scans in a darkened room. To minimize head motion, the head of the participants was fixated in the head coil using foam cushions. The scanner protocol included four functional measurements including (in this particular order) a resting‐state scan, three experimental paradigms, and a structural scan. Modality‐specific (structural MRI and resting‐state fMRI [rs‐fMRI]) findings resulting from univariate statistics are reported in Horvath et al. (2020). Results of the CR‐task employed in this study are reported in Schmitgen et al. (2020).

During acquisition of structural data, 192 T1‐weighted images were recorded, which were acquired with an MP‐RAGE pulse sequence in a transverse (axial) orientation with the following parameters: repetition time = 1900 ms, echo time = 2.52 ms, field of view = 350 × 263 × 350 mm, flip angle = 9°, voxel size = 1 × 1 × 1 mm3, 192 slices, and slice thickness = 1 mm.

For rs‐fMRI, participants were instructed to keep their eyes closed, not to fall asleep, and to not think about anything in particular. During rs‐fMRI, 200 whole brain echo planar imaging volumes were recorded in an axial orientation with the following imaging parameters: repetition time = 2000 ms, echo time = 30 ms, field of view = 192 mm, flip angle = 90°, voxel size = 3 × 3 × 3 mm3, 33 slices, and distance factor between slices = 1 mm.

### 2.3 Data preprocessing

Individual data were preprocessed as described in Horvath et al. (2020). In particular, GMV of T1‐weighted sMRI images was calculated using the Computational Anatomy Toolbox, CAT12 (http://dbm.neuro.uni‐jena.de/cat/) together with SPM12 (http://www.fil.ion.ucl.ac.uk/spm). Preprocessing included segmentation of images into gray matter, white matter, and cerebrospinal fluid, normalization using DARTEL, and smoothing of GMV segments using an 8‐mm full‐width half‐maximum (FWHM) isotropic Gaussian kernel.

Rs‐fMRI images were processed using the Data Processing Assistant for rs‐fMRI (DPABI/DPARSF) (Chao‐Gan & Yu‐Feng, 2010). Preprocessing included slice timing, head motion correction, spatial normalization (Montreal Neurological Institute (MNI) space; voxel size 3 × 3 × 3 mm), smoothing with a 4‐mm FWHM isotropic Gaussian kernel, regressing out nuisance covariates including mean signals from white matter and cerebrospinal fluid as well as the Friston 24‐parameter model (Friston et al., 1996). Afterward, ALFF‐maps were calculated based on the preprocessed data.

### 2.4 Parallel ICA

P‐ICA (J. Liu et al., 2009) on GMV and ALFF data was applied using the Fusion ICA Toolbox (FIT; version 2.0d; https://trendscenter.org/software/fit/) implemented in MATLAB 9.4.0(R2018a). The number of components for each modality was estimated using the minimum description length (MDL) and the Akaike information criterion, as described in Calhoun et al. (2001). Four components were identified for GMV and five components were identified for ALFF. ICASSO (Himberg et al., 2004) was used to assess results after running the approach 20 times to ensure consistency of the components.

For component visualization, the source matrix was reshaped back to a 3D‐image, scaled to unit standard deviations (z), and a threshold of *z* > 3 was applied. Maps from the two components described in Section 3 were overlaid onto an MNI normalized anatomical template. Anatomical denominations and stereotaxic coordinates were derived from clusters above a threshold of *z* > 3 by linking the ICA output images (i.e., the chosen components of interest) to the Talairach Daemon data base (http://www.talairach.org/daemon.html).

### 2.5 Statistical analysis

A first component preselection was performed by two sample *t*‐tests on network strength indices, that is, loading coefficients of each component, as implemented in FIT at *p* < .1. Three components fulfilled this criterion (one GMV and two ALFF) and loading coefficients of these components were extracted to be included in three analysis of covariance (ANCOVA) models to test for group differences (adjusted for age and gender). The false discovery rate (FDR) was applied to adjust *p*‐values for multiple comparisons of the three ANCOVA models, and components of interest were defined as models showing an FDR‐corrected *p*‐value of < .05. Hereby, two components of interest (both ALFF‐based) were identified.

The component loadings of the two identified components of interest were used to test for associations with SPAI‐I factors and SPAI‐I total score by partial correlations (adjusted for age and gender). FDR‐correction was applied, and results were defined significant, if the FDR‐corrected *p*‐values were < .05. ANCOVA models and partial correlations were computed in R (version 3.6.1; https://cran.r‐project.org/).

## 3 RESULTS

### 3.1 Demographic and psychometric data

Demographic and psychometric details of the two groups are given in Table 1. The groups differed significantly in BDI, BIS‐attentional score, BIS‐motor score, SAS, SPAI scores, and SPAI‐I scores (see Table 1).

**TABLE 1:** Demographics and psychometric scores

	SPA (mean)	SD	Min–Max	n‐SPA (mean)	SD	Min–Max	Statistic (df)	*p*	Effect size
Sample size	20	–	–	24	–	–	–	–	–
Age	22.20	3.04	18–28	23.00	3.26	19–30	203.5^a^	>.05	−0.13^e^
Gender (m/f)	6/14	–	–	7/17	–	–	0.004 (1)^b^	>.05	0.009^f^
BDI total score	9.85	7.10	1–25	4.38	4.20	0–15	364^a^	**.003**	−0.44^e^
BIS attentional score	17.15	3.48	11–26	13.67	2.55	10–19	−3.83 (42)^c^	**<.001**	1.16^g^
BIS motor score	22.25	3.27	17–30	20.42	3.09	17–29	326.5^a^	**.04**	−0.313^e^
BIS nonplanning score	25.40	4.43	18–34	23.17	2.97	18–30	1.99 (42)^c^	>.05	0.60^g^
SAS total score	40.65	5.73	31–51	21.71	4.08	14–28	−12.77 (42)^c^	**<.001**	3.87^g^
SPAI total score	56.85	10.03	38–82	35.75	6.24	27–55	462^a^	**<.001**	−0.79^e^
SPAI withdrawal	15.60	3.86	8–20	9.46	2.70	7–19	427.5^a^	**<.001**	−0.67^e^
SPAI compulsive behavior	17.65	3.56	13–25	11.79	2.15	9–18	6.45 (29.99)^d^	**<.001**	2.04^g^
SPAI tolerance	7.65	2.01	4–12	4.46	1.47	3–8	431^a^	**<.001**	−0.68^e^
SPAI functional impairment	15.95	3.73	10–26	10.04	2.46	8–15	440^a^	**<.001**	−0.72^e^
SPAI‐I total score	52.80	9.70	34–78	32.38	5.20	25–48	−8.46 (27.86)^d^	**<.001**	2.70^g^
SPAI‐I time spent	10.75	2.02	6–13	5.92	1.41	4–11	454^a^	**<.001**	−0.77^e^
SPAI‐I compulsivity	6.50	2.16	4–12	4.42	0.72	4–6	384^a^	**<.001**	−0.55^e^
SPAI‐I daily life interference	14.65	3.87	10–27	9.54	1.44	8–14	455^a^	**<.001**	−0.77^e^
SPAI‐I craving	13.45	3.20	7–19	8.38	2.75	5–15	−5.64 (42)^c^	**<.001**	1.71^g^
SPAI‐I sleep interference	7.45	2.21	4–12	4.13	1.98	3–10	422^a^	**<.001**	−0.66^e^
IGDS‐sf total score	0.65	1.09	0–4	0.42	0.72	0–2	259^a^	>.05	−0.08^e^

*Note*: Significant results are highlighted in bold font. Abbreviations: BDI, Beck Depression Inventory; BIS, Barratt Impulsiveness Scale; df, degrees of freedom; f, female; IGDS‐sf, short‐form of the Internet Gaming Disorder Scale; m, male; n‐SPA, non‐SPA (controls); SAS, Smartphone Addiction Scale; SD, standard deviation; SPA, addictive smartphone use group; SPAI, Smartphone Addiction Inventory; SPAI‐I, five‐factor solution of the SPAI.

^a^ Wilcoxon rank sum test.

^b^ *χ* ^2^.

^c^ Two sample *t*‐test.

^d^ Welch two sample *t*‐test.

^e^ r.

^f^ φ.

^g^ Cohen's *d*.

### 3.2 Parallel ICA

**FIGURE 1:**
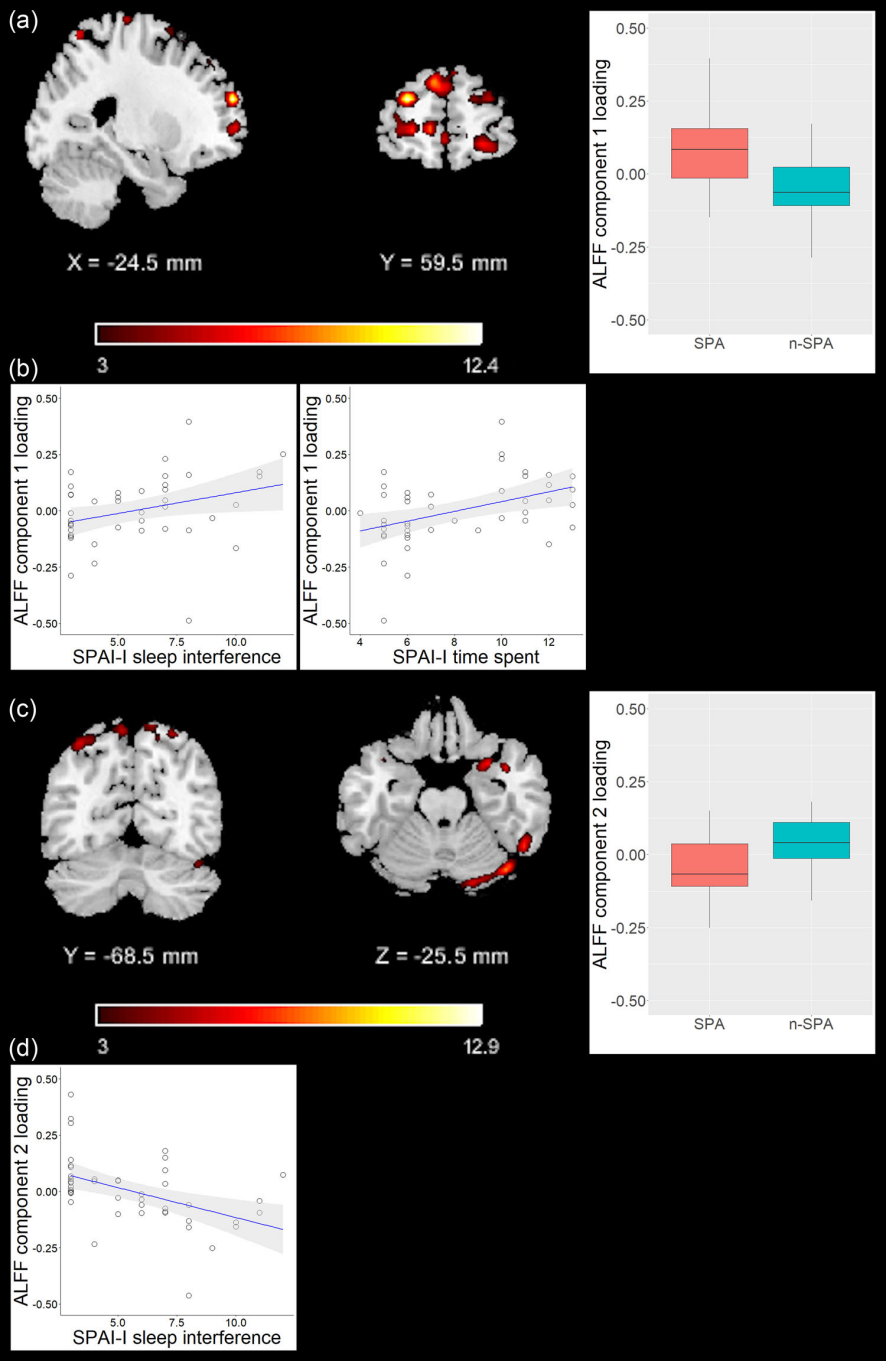
Visualization of amplitude of low frequency fluctuations (ALFF) components’ localizations, component loadings, and correlations. (a) Left, middle: overlay of ALFF 1 component pattern onto a brain template, X and Y show coordinate of the respective slice, color bar depicts *z*‐values. Right: boxplot of ALFF 1 component loadings by group (*p*_FDR_ = .003; analysis of covariance (ANCOVA), adjusted for age and gender). (b) Scatter plots of ALFF 1 component loadings versus SPAI‐I factor scores. (c) Left, middle: overlay of ALFF 2 component pattern onto a brain template, Y and Z show coordinate of the respective slice, color bar depicts *z*‐values. Right: boxplot of ALFF 2 component loadings by group (*p*_FDR_ = 0.025; ANCOVA, adjusted for age and gender). (d) Scatter plot of ALFF 2 component loadings versus SPAI‐I factor score. Shaded area around blue regression line depicts 95% confidence interval. Abbreviations: SPA: addictive smartphone use group, n‐SPA: non‐SPA

Components preselected subsequently were entered in ANCOVA models to test for group differences. The first ALFF‐based component (ALFF 1) predominantly comprised medial and dorsolateral prefrontal regions and also included temporal and parietal regions. The second ALFF‐based component (ALFF 2) predominantly comprised parietal and cerebellar regions and also included frontal, temporal, and occipital regions (see Table 2, Figure 1). ANCOVA models revealed significant differences of component loadings between groups for ALFF 1 and ALFF 2, but not for GMV (ALFF 1: *F* = 12.85, df = 1, *p*_FDR_ = .003, median_SPA_ = 0.83, median_n‐SPA_ = −0.06; ALFF 2: *F* = 5.41, df = 1, *p*_FDR_ = .038, median_SPA_ = −0.07, median_n‐SPA_ = 0.04; GMV: *F* = 3.21, df = 1, *p*_FDR_ = .081, median_SPA_ = 0.03, median_n‐SPA_ = −0.04). ALFF 1 and ALFF 2 were therefore identified as components of interest to be used for subsequent correlation analyses with distinct SPAI‐I dimensions. For completeness, regions comprised by the GMV component are depicted in Table S1.

**TABLE 2:** Spatial characteristics of identified components of interest

Component	Brodmann area	L	R	Volume (cc) L/R
region		*z*‐Score/MNI (x, y, z)	*z*‐Score/MNI (x, y, z)	
ALFF 1				
Superior frontal gyrus	6, 8, 9, 10, 11	12.0 (−24, 60, 21)	6.6 (30, 27, 57)	4.7/2.6
Medial frontal gyrus	9, 10	7.9 (−9, 63, 3)	6.4 (6, 66, 9)	1.7/1.2
Middle frontal gyrus	6, 8, 9, 10, 46	7.2 (−21, 60, 24)	4.5 (33, 60, −9)	2.0/0.7
Superior temporal gyrus	22, 38	7.1 (−54, 15, −9)	5.5 (57, 15, −3)	0.6/0.8
Inferior frontal gyrus	10, 47	6.5 (−54, 18, −6)	5.8 (54, 18, −3)	0.4/0.8
Precuneus	7	5.5 (−6, −81, 45)	5.4 (18, −75, 51)	0.4/1.2
Middle temporal gyrus	37	–	4.0 (66, −33, −12)	‐/0.3
Precentral gyrus	6	5.1 (−36, −18, 66)	–	0.6/‐
Postcentral gyrus	1, 2, 5, 7	–	5.1 (51, −27, 60)	‐/0.8
Superior parietal lobule	7	4.9 (−12, −63, 63)	4.3 (36, −69, 51)	0.6/0.3
Inferior parietal lobule	7, 40	4.0 (−60, −27, 30)	–	0.4/‐
ALFF 2				
Superior frontal gyrus	6, 8, 9, 10	7.4 (−15, 42, 51)	9.2 (33, 54, 21)	2.9/4.2
Declive	–	–	9.0 (42, −75, −27)	‐/1.3
Tuber	–	–	8.5 (57, −51, −30)	‐/0.4
Superior temporal gyrus	38	–	8.2 (42, 6, −24)	‐/0.5
Uncus	28	–	7.7 (30, 9, −24)	‐/0.3
Lingual gyrus	17, 18	5.5 (−3, −93, −6)	7.6 (12, −87, −21)	0.4/0.7
Middle frontal gyrus	6, 8, 9, 10, 46	7.1 (−30, 54, 24)	7.5 (36, 51, 24)	2.8/3.5
Cuneus	17, 18, 19	–	7.0 (6, −99, 3)	‐/1.3
Fusiform gyrus	18, 19, 37	–	6.3 (21, −87, −21)	‐/0.4
Inferior frontal gyrus	9, 45, 46, 47	5.1 (−48, 36, 15)	4.4 (27, 9, −21)	0.4/0.3
Medial frontal gyrus	6, 9, 10	4.8 (−6, 54, 42)	5.1 (3, 60, 12)	0.5/1.3
Superior parietal lobule	7	4.8 (−36, −69, 51)	–	0.6/‐
Inferior parietal lobule	7, 40	4.6 (−48, −54, 54)	–	0.3/‐

Voxels with *z* > 3 were coupled with the Talairach Daemon database to provide anatomical labels and were translated into MNI space. For each hemisphere (L = left; R = right), the maximum *z*‐value and MNI coordinate are provided. The volume of voxels in each area is provided in cubic centimeters (cc); the table displays clusters > 0.2 cc.

### 3.3 Associations between functional networks and SPAI‐I

Loading parameters of both components correlated with SPAI‐I total score (ALFF 1: *p*_FDR_ = 0.04, correlation coefficient = 0.38; ALFF 2: *p*_FDR_ = .03, correlation coefficient = −0.38; see Table 3). Particularly, loading parameters of ALFF 1 correlated with SPAI‐I time spent (*p*_FDR_ = .03, correlation coefficient = 0.45) and SPAI‐I sleep interference (*p*_FDR_ = .04, correlation coefficient = 0.38), and loading parameters of ALFF 2 correlated with SPAI‐I sleep interference (*p*_FDR_ = .02, correlation coefficient = −0.46; see Table 3). For completeness, correlations between component loadings of the GMV component and SPAI factors are depicted in Table S2.

**TABLE 3:** Partial correlations between amplitude of low frequency fluctuations (ALFF) component loadings and Smartphone Addiction Inventory (SPAI‐I) measures

Component	SPAI‐I measure	*p*_FDR_	Correlation coefficient
ALFF 1	SPAI‐I total score	**0.04**	0.38
ALFF 1	SPAI‐I time spent	**0.03**	0.45
ALFF 1	SPAI‐I compulsivity	0.18	0.21
ALFF 1	SPAI‐I daily life interference	0.09	0.27
ALFF 1	SPAI‐I craving	0.07	0.29
ALFF 1	SPAI‐I sleep interference	**0.04**	0.38
ALFF 2	SPAI‐I total score	**0.03**	−0.38
ALFF 2	SPAI‐I time spent	0.09	−0.29
ALFF 2	SPAI‐I compulsivity	0.06	−0.32
ALFF 2	SPAI‐I daily life interference	0.06	−0.32
ALFF 2	SPAI‐I craving	0.15	−0.23
ALFF 2	SPAI‐I sleep interference	**0.02**	−0.46

*Note*: Partial correlations for all subjects (*n* = 44). Adjusted for age and gender. *p*_FDR_ provides FDR‐corrected *p*‐values. Significant results (*p*_FDR_ < .05) are highlighted in bold font.

Complementary analyses additionally adjusted for BDI total score showed no significant associations between loading parameters (ALFF 1, ALFF 2, and GMV) with SPAI‐I or SPAI factors or respective total score.

SPAI‐I time spent and SPAI‐I sleep interference were correlated (*p* < .001, correlation coefficient = 0.60), also with an additional adjustment for BDI total score (*p* < .001, correlation coefficient = 0.51).

## 4 DISCUSSION

We used a multivariate data‐driven approach to study associations of two modalities, that is, functional and structural imaging findings in the context of addictive smartphone use. Two key findings emerged: First, a predominantly medial/dorsolateral prefrontal and a mainly parietal/cerebellar functional network differed significantly between SPA and n‐SPA. Second, there were specific and shared correlations between these functional systems and dimensional measures of addictive smartphone use.

Our analyses identified two rs‐fMRI components, which differed between SPA and n‐SPA. The first component showed increased activation in predominantly medial and dorsolateral prefrontal regions in those with SPA versus n‐SPA. In IGD, a diagnosis showing substantial conceptual overlap to SPA (Paik et al., 2017), common neurobiological mechanisms, particularly with respect to prefrontal cortex among other regions, are suggested (Kuss et al., 2018). The second component showed decreased activation in predominantly parietal and cerebellar regions in those with SPA versus n‐SPA. Cerebellar function has been associated with habit‐formation in addictive disorders (Noori et al., 2016). Therefore, it is conceivable that the found group‐difference of cross‐information in this component may reflect a link to habit formation in excessive smartphone use. Nevertheless, specifically tailored studies are needed to test this hypothesis, preferably study designs that complement resting‐state data acquistion with specific experimental tasks.

On a neural systems level, regions depicted by the two functional networks are part of the so‐called “default mode network” (DMN), which has been associated to self‐awareness in addictive disorders and the salience network, which critically mediates the interplay between executive systems and the DMN (Volkow et al., 2012). Moreover, the detected patterns share, if at all, only little spatial similarities reported by Horvath et al. (2020). This indicates that the multivariate fusion approach used here indeed revealed information, which was not detected via separate analyses of GMV and INA.

Remarkably, the functional networks also showed significant correlations with distinct SPAI‐I dimensions, that is, “time spent” (particularly the medial/dorsolateral prefrontal system) and “sleep interference” (both networks). Noteworthy, these SPAI‐I dimensions were associated with each other in our sample. The two identified components share the same imaging modality, that is, rs‐fMRI, yet they reflect different physiological properties and cognitive representations on a subnetwork‐level. Moreover, nonfusion ICA‐based methods on ALFF alone very likely would have not been able to identify the found components without the additional information from GMV. In this line of thought, the found correlations depict component specific and shared associations between temporal features of intrinsic neural activity and measures of addictive smartphone use. Most of the regions found in the prefrontal networks are part of the inhibitory networks depicted by Stevens et al. (2007); therefore, the found associations might represent a lack of inhibitory control in SPA. This notion is also supported by the significant differences between SPA and n‐SPA with respect to BIS attention and motor scores. The SPAI‐I factor sleep interference includes items of the SPAI that capture the relationship between smartphone use and shorter sleep duration as well as sleep disturbance (Pavia et al., 2016). Regions found in both networks are part of the DMN, which represents brain activation during a relaxed, resting condition without external stimuli (Raichle et al., 2001). Altered functional activation in these regions might represent, among others, rumination processes and self‐awareness, as reported for resting‐state functional connectivity in substance‐based addictions using group‐ICA‐based methods (Zhang & Volkow, 2019)—or even excessive preoccupation with the smartphone, which in turn disrupts healthy sleeping behavior leading to depressed mood (Wacks & Weinstein, 2021; Slavish & Graham‐Engeland, 2015). Further, DMN involvement (van der Linden et al., 2021) may point to a biological basis of “flow” theories in internet addiction (Thatcher et al., 2008; Stavropoulos et al., 2018). Online flow may be implicated in the experience of those with SPA, alongside other psychological mechanisms (i.e., fear of missing out), supporting the maintenance of the behavior, as previously discussed (Fauzi et al., 2021). Taken together, these findings point toward a multifaceted, brain‐activation linked interplay of noncapability of not using the smartphone and sleep interfering processes in SPA.

We acknowledge potential limitations of this study, such as the relatively small sample size and missing formal clinical evaluation of potentially confounding comorbid mental disorders. Mental disorders were not reported by the participants, but it is impossible to fully rule out the presence of other mental health conditions that may have an impact on GMV or ALFF. Moreover, the identified associations between loading parameters and measures of SPA did not remain significant, if additionally controlled for depressiveness. The detected relationships between SPAI‐I time spent and SPAI‐I sleep interference remained significant after BDI adjustment, indicating that depressive symptoms may not fully account for such associations. Therefore, our interpretations of these findings should be handled with appropriate scientific caution, and future studies with a tailored design are needed to clarify the role of depressiveness in SPA and its dimensions. Our hypothesis about a possible impairment of cognitive control mechanisms is only based on structural and functional resting‐state MRI. Future studies should complement these modalities by a broad array of specific cognitive tasks in order to establish convincing brain‐behavior relationships. Additionally, the cross‐sectional design does not allow inference on temporal development and stability of these findings, as much as the correlations do not imply causality. Carefully designed longitudinal studies with sample sizes being more representative of the population showing problematic smartphone use are needed to robustly answer such important questions.

In conclusion, the present study provides further evidence for common neural mechanisms underlying technology‐related behavioral addictions, including SPA. The findings of this study depict a fronto‐parietal network, which has been previously related to disrupted cognitive control in internet addiction (Wang et al., 2017). Furthermore, the data support the notion of a disbalance between systems subserving prefrontal control, as suggested by the I‐PACE model (Brand et al., 2016). This study needs replication and extensions using larger and more representative sample sizes, including longitudinal assessments supplemented by ecological momentary assessment. Additionally, this study provides important new findings, suggesting deviant recruitment of resting‐state networks and modality‐specific associations of resting activation with distinct and shared symptom dimensions of SPA.

## CONFLICT OF INTEREST

The authors declare no conflict of interest.

### PEER REVIEW

The peer review history for this article is available at: https://publons.com/publon/10.1002/brb3.2739.

## Supporting information

Table S1 Spatial characteristics of the GMV component.
Table S2 Partial correlations between component loadings and behavioral measures.

## Data Availability

The data that support the findings of this study are available from the corresponding author upon reasonable request.
